# Effect of ambient air pollution on premature SGA in Changzhou city, 2013–2016: a retrospective study

**DOI:** 10.1186/s12889-019-7055-z

**Published:** 2019-06-07

**Authors:** Shushu Li, Huaiyan Wang, Haiting Hu, Zeying Wu, Kejin Chen, Zhilei Mao

**Affiliations:** 1 Changzhou Center for Disease Control and Prevention, Changzhou, 213022 Jiangsu China; 2 Changzhou Maternity and Child Health Care Hospital affiliated to Nanjing Medical University, Changzhou, 213003 Jiangsu China; 3 Changzhou Institute of Technology, Changzhou, 213003 Jiangsu China; 4 State Key Laboratory of Reproductive Medicine, Center for Global Health, Nanjing Medical University, 101 Longmian Road, Nanjing, 211100 China; 5 Key Laboratory of Modern Toxicology of Ministry of Education, School of Public Health, Nanjing Medical University, 101 Longmian Road, Nanjing, 211100 China

**Keywords:** Air pollution, Premature SGA, Exposure windows, Retrospective study

## Abstract

**Background:**

Air pollution is becoming an increased burden to the world. Previous studies have confirmed its effects on adverse birth outcomes, but few associated with premature small for gestational age (SGA). We report a retrospective cohort study conducted in Changzhou city to evaluate the association between air pollutants (PM_2.5_, SO_2_ and NO_2_) and premature SGA during pregnancy.

**Methods:**

A total of 46,224 births were collected from January, 2013 to December, 2016, in Changzhou Maternity and Child Health Care Hospital, finally 2709 preterm births were admitted for study. Corresponding air monitoring data were collected from Changzhou Environmental Protection Agency. Generalized estimating equations were used to examine the association between these air pollutants and premature SGA controlling for individual covariates in single- and multi-pollutant models.

**Results:**

We found that, in the third trimester, every 10 μg/m^3^ increments in PM_2.5_ concentration were associated with premature SGA (OR = 1.18, 95% CI: 1.03–2.83; OR = 1.37, 95% CI: 1.03–3.58) in two- and three-pollutants models. In the whole gestation, a 10 μg/m^3^ increment in PM_2.5_ concentration in two- and three-pollutant models were related to premature SGA (OR = 1.53, 95% CI: 1.38–2.47; OR = 1.73, 95% CI: 1.18–2.57). The OR (95% CI) of premature SGA were increasing across quintiles of PM_2.5_, SO_2_, NO_2_ concentrations during the whole gestation period adjusting for confounders (*P*_for trend_ < 0.001).

**Conclusions:**

These results indicated that pregnant women exposed to PM_2.5,_ combined with other pollutants in the third trimester have a higher risk to deliver premature SGA babies, providing further evidence linking PM_2.5_ and pregnancy outcomes.

## Background

With the rapid development of economy, air pollution has become a serious public health problem, and gained much global attention. A variety of studies have revealed the association of air pollution with many human diseases, such as respiratory infections [1], cardiovascular diseases [2], Parkinson’s disease [3], and depression [4], These diseases generated a large burden of mortality and years of life lost [5, 6]. More recently, an increasing number of researches [7, 8] have indicated the potential associations between air pollutants exposure and the adverse pregnancy outcomes and mortality. Generally, adverse birth outcomes and fetal health during pregnancy could be evaluated by some common indicators including growth restriction, also known as small for gestational age (SGA), which was defined as a birth weight below the 10th percentile for the same gestational age by sex; preterm birth (PTB), defined as a live birth before the 37th week of gestation; and low birth weight (LBW), defined as a birth weight less than 2500 g. Air pollutants were reported had adverse impacts on fetal development in both animal and human studies [9, 10]. Epidemiological studies mainly focused on PTB [11–13], SGA [14] and LBW [15–17]. A literature from [18] reviewed the association between air pollutants (including particulate matters below 10 and 2.5 μm in aerodynamic diameter (PM_10_ and PM_2.5_), nitrogen dioxide (NO_2_), sulphur dioxide (SO_2_), and carbon monoxide (CO)) and adverse birth outcomes (low birth, LBW and PTB). Another recent research [19] indicated that exposure to high concentrations of PM_2.5_ in the second trimester and exposure to PM_10_ in the late pregnancy had a strong effect on PTB. Le et al. [20] also found that SGA was associated with CO levels exceeding 0.75 ppm and NO_2_ exceeding 6.8 ppb exposure in the first month. Although some studies had confirmed the significantly impacts of air pollutants on PTB and SGA, few studies had elucidated the relationship between air pollutants and premature SGA. However, whether they would affect the premature SGA is still unclear.

Therefore, this study set to address this important question. It has been realized that the nature of the components in particulate matters mainly depend on local natural environment, industrial types and meteorological conditions, and that different composition would impose different effects [21]. Given that the PM_2.5_ components differ among different regions, local studies could not be replaced by those performed in other regions. In addition, the genetic variation of different populations is known to be associated with different responses to the environmental risks [22], so the European or African population studies reported may not represent the situation in the Asia population.

In this study, we recruited only the premature birth and analyzed the associations between premature SGA and air pollutions in Changzhou city, and excluded other factors which may affect premature on SGA.

## Methods

### Study population

Our study subjects were obtained from a retrospective study of the premature infants and their mothers from Changzhou Maternity and Child Health Care Hospital affiliated to Nanjing Medical University during 2013–2016. Women below 37 weeks of gestational durations were enrolled in the birth registry. Birth records included the data of infant gender, date of birth, gestational age, birth weight, birth order, number of stillborn (if multiple births), maternal age at child’s birth, total number of live born and stillborn (ever). Maternal behaviors including smoking and alcohol consumption were not available. Small for gestational age (SGA) were defined as *<*10th percentile of birth weight of gestational age. The first trimester of pregnancy was defined as gestational week 1 to 12, the second trimester as week 13 to 27 and the third trimester as from week 28 to birth.

### Exposure assessment

In this study, daily air pollutants (PM_2.5_, SO_2_ and NO_2_) concentrations were simultaneously collected from seven fix-sited air pollution monitoring stations in Changzhou, including Chengjian school, Anjia, Wujin, Lucheng, Changgongyuan and Changzhou city from January 1, 2013 to December 31, 2016 in Changzhou Environment Protection Agency. The inverse distance weighting (IDW) method was performed by a geographic information system (ARCGIS, version 10.3; ESRI, Redlands, CA, USA) according to the following procedures. First, the residential address of each pregnant women registered in the medical records was transferred into longitude and latitude using xGeocoding software. Researchers indicated the monitoring data could represent a good indicator of individual exposure if subjects resided less than 40 km from the nearest station. And the minimum and maximum distance from individuals’ residence addresses to the nearest monitoring station were measured at 0.04 and 29.44 km, respectively, in our study. Then, an appropriate spatial resolution (100 m) was used to divide study areas into 100 m × 100 m grid cells (Rivera-González et al., 2015). Finally, we used the inverse squared distance (1/squared distance) to weight the monitoring data of the nearest station and the coordinates of subjects were applied to calculate the daily pollution levels. The gestational age and birth date were used to calculate trimester-specific and entire pregnancy periods. The air pollution measurements were integrated into monthly summaries.

We used an inverse distance weighting approach to predict residential sites across Changzhou. For each birth, the pregnancy and trimester-specific pollution exposure measurements were calculated by monthly summaries. Data from the six monitoring stations were included in each interpolation. At last, we counted the average value of individual exposure level in each trimester and whole pregnancy.

### Covariates

Individual characteristics in the analyses included maternal age at delivery (categorized into *<* 20, 20–30, 30–40, *>* 40 years old), gestational age (continuous), parity (the numbers of pregnancy, once or more than once), natural delivery or not, sex of infants (male or female), birth years (from 2013 to 2016). Birth seasons were classified into Spring: March – May; Summer: June – August; Fall: September – November; and Winter: December–February. Meteorological information, including mean temperature, relative humidity and air pressure, were obtained from the local department.

### Statistical analysis

For descriptive analysis, the min, max, mean, standard deviation (SD), median, percentiles and interquartile range (IQR) were calculated for air pollutants. The correlation among different air pollutants concentrations were assessed by Spearman correlation coefficient. Potential confounders of the relationships between air pollution exposure and premature SGA were described in terms of mean (quantitative variables) or percentages (qualitative variables). In order to adjust for correlations within people belonging to the same city, a generalized estimating equations (GEE) models were used to examine the adjusted odds ratios (ORs) and 95% confidence intervals (CIs) for premature SGA and air pollutants in each exposure windows, adjusting for covariates including infant sex, gestational age, maternal age, parity, methods of delivery, season of birth, year of birth and meteorological information. Before running model, the linearity and independence of variants were checked by Durbin-Watson and scatter plots. The multi-collinearity was tested using variance inflation factor (VIF) which was less than five for all of the independent variables. The concentration-response curves were drawn using restricted cubic spline regression model. All statistical analyses were conducted using R3.3.1 (RCorp., College Station, TX, USA).

## Results

**Fig. 1:**
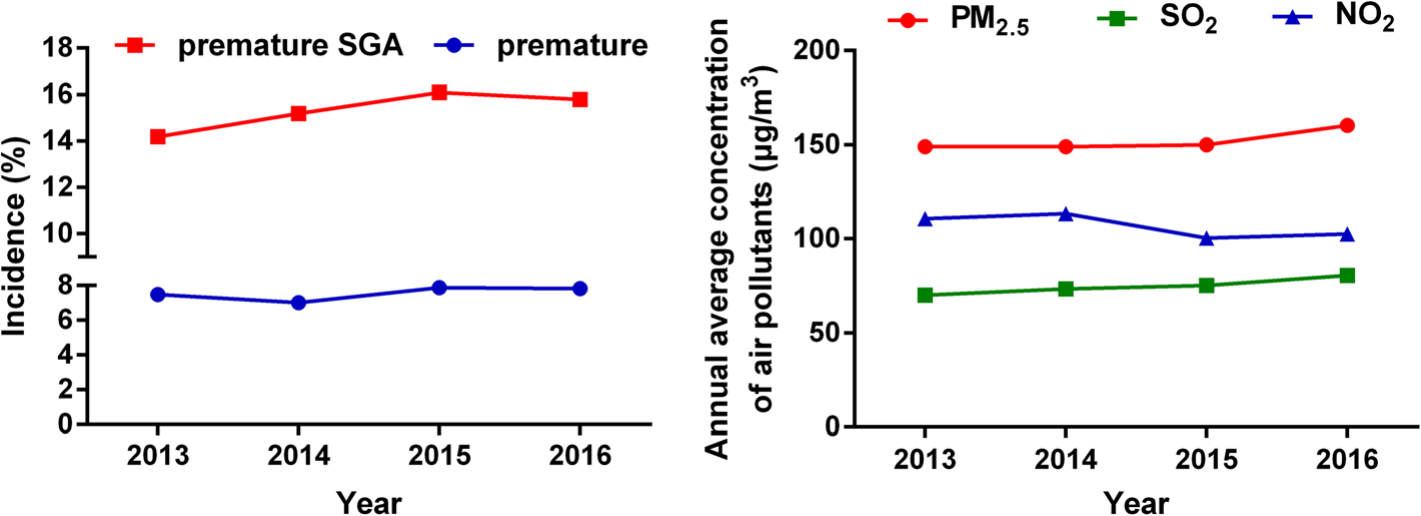
The median concentrations of air pollutants and incidences of premature SGA from 2013 to 2016. SGA: small for gestational age, PM2.5: particular matter 2.5, NO_2_: nitrogen dioxide, SO_2_: sulphur dioxide

**Fig. 2:**
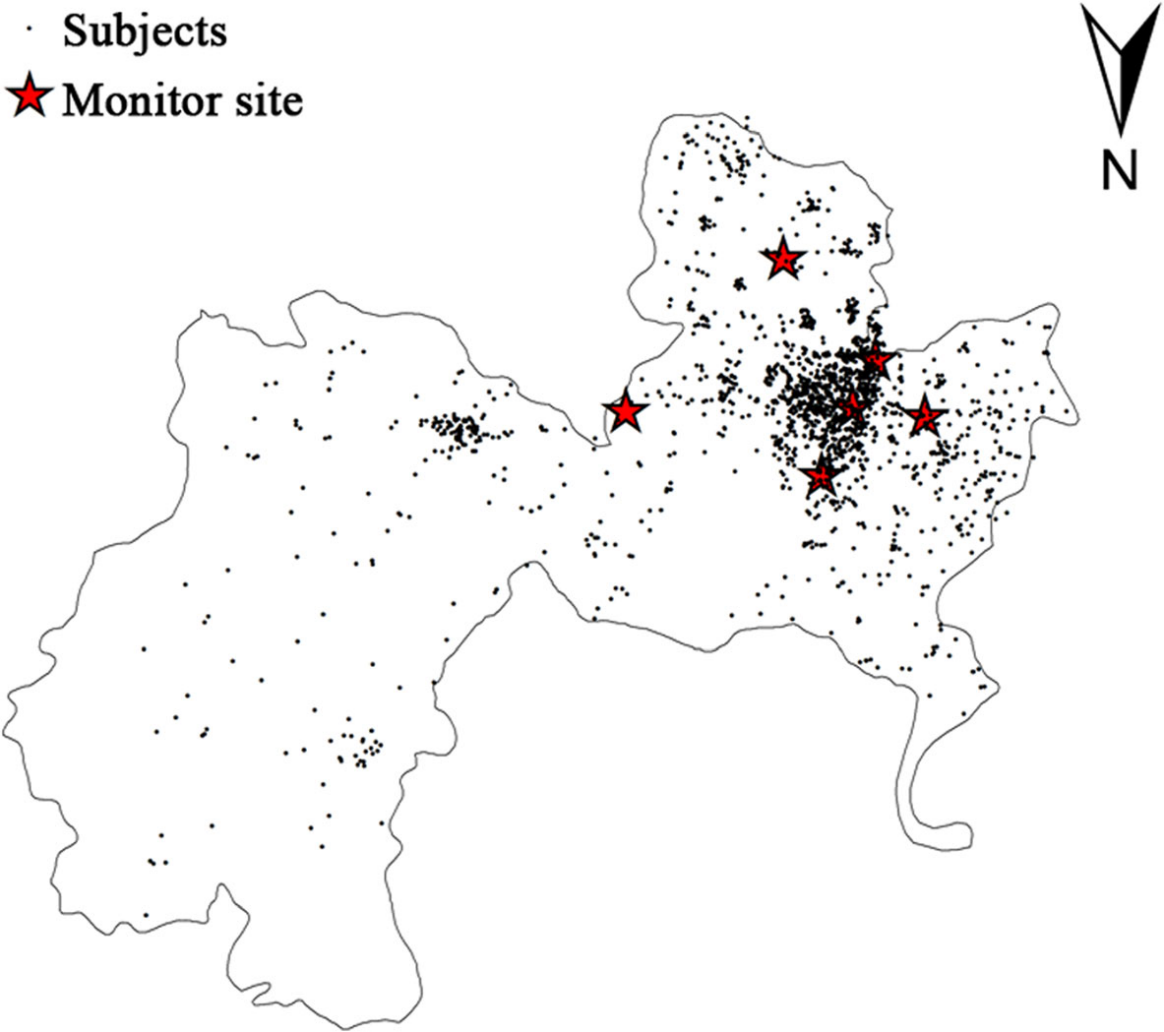
Location of monitoring sites and subjects. Six monitoring sites and 2709 pregnant women with premature births were marked by red pentagrams and black solid dots, respectively. The map was generated by the professional software (ARCGIS, version 10.3; ESRI, Redlands, CA, USA)

During the study period, there were 10,028 (2013), 12,198 (2014), 10,743 (2015), and 13,255 (2016) live births, in which 750 (2013), 856 (2014), 847 (2015), and 1078 (2016) preterm births were enrolled from the retrospective cohort study. There are 107 (2013), 130 (2014), 136 (2015), and 170 (2016) preterm SGA. The overall incidence of premature was 7.48, 7.02, 7.88, 7.83% and premature SGA was 14.2, 15.2, 16.1, 15.8%, both of which were increased with years (Fig. 1). After further excluding the births with missing birth records and covariate data, 2709 preterm births were admitted and analysis of preterm SGA were based on 479 births. As Fig. 2 showed, the spatial distribution of seven air quality monitoring stations and residence addresses of 2709 premature births is were marked by black solid dots and red pentagrams, respectively, during 2013–2016.

Table 1 shows the summary statistics of air pollutants from 2013 to 2016. Compared to the air quality guidelines issued by World Health Organization (WHO), in which the standard of PM_2.5_, SO_2_ and NO_2_ are 25 mg/m^3^, 20 mg/m^3^, and 44 mg/m^3^, respectively, their corresponding average concentrations in Changzhou were 2.0, 1.3 and 0.8 times higher during the whole pregnancy period.

**Table 1:** Summary of estimated air pollutants by exposure period

Pollutant (μg/m^3^)	Mean	SD	Percentile							
			Min	5th	25th	50th	75th	95th	Max	IQR ^b^
PM_2.5_										
Trimester1	39.80	31.82	5.72	30.95	41.12	53.97	66.88	77.58	98.43	25.76
Trimester2	53.63	20.68	9.08	37.42	49.29	60.31	66.91	78.51	90.65	17.61
Trimester3	59.98	15.62	7.19	36.34	46.15	61.43	70.79	83.17	95.17	24.64
Whole gestation	51.11	16.03	12.32	25.67	37.40	53.75	63.85	74.19	86.94	26.45
SO_2_										
Trimester1	21.37	16.57	8.52	12.37	16.45	28.41	34.88	42.25	50.57	18.43
Trimester2	26.21	10.50	10.88	15.86	22.50	28.96	33.10	38.32	48.01	10.60
Trimester3	28.82	8.27	10.05	16.10	23.44	30.33	34.04	41.69	48.31	10.59
Whole gestation	25.46	8.52	5.44	11.31	18.82	26.45	32.72	37.38	45.51	13.90
NO_2_										
Trimester1	28.03	22.30	9.24	21.85	32.00	40.09	46.68	52.05	78.89	14.68
Trimester2	34.62	14.99	10.07	12.63	24.40	39.91	45.50	50.79	57.13	21.10
Trimester3	43.08	7.24	10.02	33.37	39.96	43.95	46.99	52.33	57.47	7.03
Whole gestation	35.23	10.50	11.47	15.33	27.95	38.12	43.72	49.12	57.44	15.77

SD: standard deviation, Min: minimum; Max: maximum, IQR: interquartile range

PM_2.5_: particular matter 2.5, NO_2_: nitrogen dioxide, SO_2_: sulphur dioxide

The incidence of premature SGA and mean PM_2.5_, SO_2_, NO_2_ exposure by infant gender, maternal age, and other characteristics are shown in Table 2. Average birth weight was 2211.8 g (SD = 522.32 g). The majority of the study population is between 20 and 40 years old (94.7%). Seasons of birth were roughly distributed among the four seasons equally. Significant differences in SGA incidence were noted by infant gender, maternal age, delivery and parity. The seasonal levels of air pollutants exhibited to be highest in winter and lowest in summer.

**Table 2:** Descriptive statistics of SGA and mean exposure over the entire pregnancy among characteristics of the included 2709 preterm births

Characteristic	SGA ^a^ [n (%)]	Mean PM_2.5_ (μg/m^3^)	Mean SO_2_ (μg/m^3^)	Mean NO_2_ (μg/m^3^)
gender				
male	250 (9.4)	51.22	76.55	105.75
female	189 (7.1)	50.98	76.12	105.55
*P*	0.000 *	0.849	0.725	0.354
Maternal age (years) ^b^				
< 20	16 (0.6)	48.85	72.73	105.36
20–30	296 (11.4)	51.05	76.33	105.82
30–40	112 (4.3)	51.51	76.66	104.65
> 40	7 (0.3)	50.77	75.76	108.82
*P*	0.000 *	0.290	0.458	0.666
Birth years				
2013	32 (1.2)	49.75	70.29	110.77
2014	130 (4.0)	49.70	73.64	113.37
2015	136 (4.8)	50.05	75.43	100.40
2016	141 (5.2)	53.48	80.72	102.55
*P*	0.747	0.079	0.279	0.571
Season of birth				
Spring	96 (3.5)	52.06	77.91	103.21
Summer	105 (3.9)	46.87	67.37	101.65
Fall	126 (4.7)	51.85	77.68	107.75
Winter	112 (4.1)	53.19	81.47	109.05
*P*	0.562	0.001*	0.000 *	0.000 *
Natural delivery				
No	110 (4.1)	49.43	74.13	104.31
Yes	329 (12.1)	52.42	78.10	106.71
*P*	0.000 *	0.549	0.000 *	0.127
Parity				
Once	151 (5.6)	50.68	75.43	106.23
Twice or more	288 (10.6)	51.68	77.37	105.97
*P*	0.000 *	0.368	0.715	0.729

a SGA: small for gestational age

b missing 58 subjects

* P < 0.05

The concentrations of PM_2.5_, NO_2_ and SO_2_ were relatively strongly correlated with each other, and their coefficients ranged from 0.522 to 0.905. The entire pregnancy exposures were highly correlated with the exposures in the three trimester period (Spearman’s r = 0.85–0.91, data not shown). Table 3 illustrates the distributions of each pollutant in premature SGA and control groups. All three pollutants showed higher values in premature SGA group than those in premature appropriate for gestational age (AGA) group, and showed no additional statistical significance over the entire pregnancy period.

**Table 3:** Comparison of median exposure of air pollutants over the entire pregnancy between cases and controls among the included 2709 preterm births

Air pollutants (μg/m^3^)	SGA^*^ (median, SD^*^)		*P*
	Control	Case	
PM_2.5_^*^			
Trimester1	53.97, 21.71	54.02, 22.19	0.233
Trimester2	60.27, 20.42	60.54, 20.84	0.134
Trimester3	61.32, 15.52	61.68, 16.32	0.303
Whole gestation	53.59, 15.84	55.69, 16.86	0.451
SO_2_^*^			
Trimester1	28.42, 16.49	29.49, 16.77	0.356
Trimester2	28.85, 10.56	29.62, 8.22	0.452
Trimester3	29.69, 8.22	30.38, 8.68	0.615
Whole gestation	26.45, 8.43	26.67, 8.92	0.734
NO_2_^*^			
Trimester1	39.63, 22.25	40.42, 22.38	0.493
Trimester2	39.85, 14.91	39.89, 14.77	0.547
Trimester3	44.05, 7.18	44.20, 7.72	0.265
Whole gestation	38.18, 10.41	38.27, 10.75	0.944

* SGA: small for gestational age; SD, Standard deviation, PM_2.5_: particular matter, NO_2_: nitrogen dioxide, SO_2_: sulphur dioxide

**Fig. 3:**
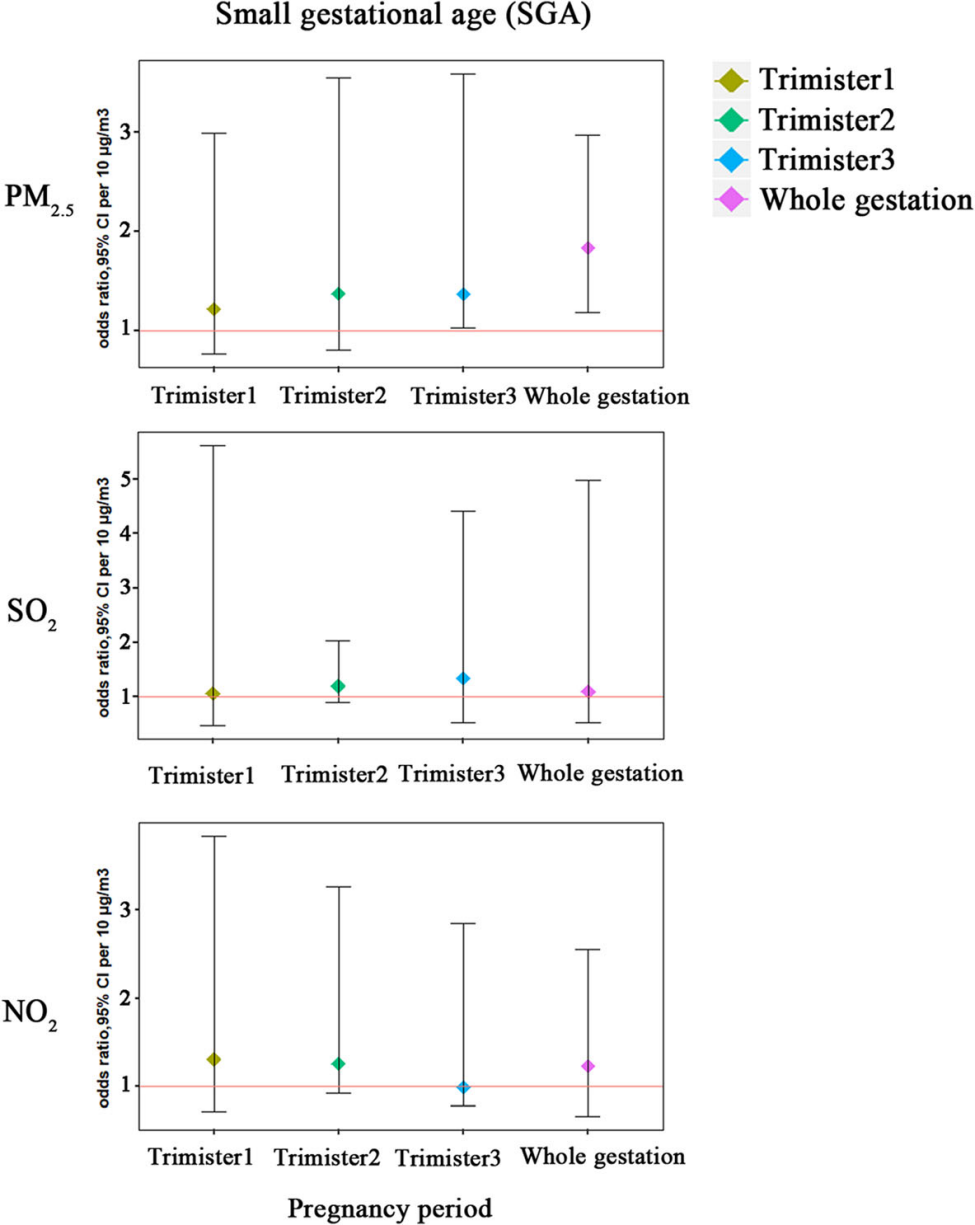
Associations of air pollutants with SGA in three-pollutants model over the entire pregnancy period. These were estimated using generalized estimating equations adjusting for infant gender, maternal age and parity, year of birth, and season of birth in SGA models, and gestational age and meteorological information (mean temperature, relative humidity and air pressure) were also included. PM_2.5_: particular matter, NO_2_: nitrogen dioxide, SO_2_: sulphur dioxide, SGA: small for gestation age

**Fig. 4:**
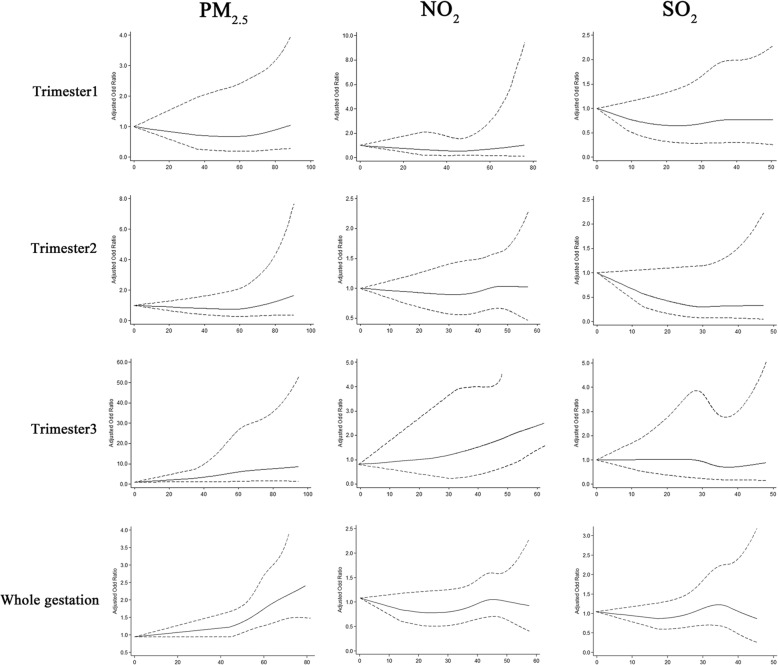
Concentration-response relationships between air pollutants and SGA in three-pollutants model over the entire pregnancy period. These were estimated using restricted cubic spline regression model adjusting for infant gender, maternal age and parity, year of birth, season of birth and meteorological information (mean temperature, relative humidity and air pressure) in multi-pollutants models. PM_2.5_: particular matter, NO_2_: nitrogen dioxide, SO_2_: sulphur dioxide, SGA: small for gestation age

Table 4 summaries the risk incidence of premature SGA in single- and multi-pollutant models for each change in pollutant concentrations during the whole pregnancy period. Figure 3 shows the associations of air pollutants with premature SGA in three-pollutant model over the entire pregnancy period, and the concentration-response curves were shown in Fig. 4. In the single-pollutant model, no significant associations were observed between premature SGA and air pollutants. The effect estimates of PM_2.5_ showed additional statistical significance when other pollutants were included in the models. There are positive associations with premature SGA for PM_2.5_ (*p* = 0.016, OR = 1.18, 95% CI:1.03–2.83; *p* = 0.009, OR = 1.37, 95% CI:1.03–3.58, respectively) in the third trimester after adjusting confounders in two- (combined with exposure of SO_2_) and three-pollutant (combined with exposure of SO_2_ and NO_2_) models, in which the relative increment in risk incidence of premature SGA for each 10 μg/m^3^ increment in PM_2.5_ concentrations were 18 and 37%, respectively during the third trimester. While in other two pregnancy periods, no significant adverse effect results were observed. In the whole gestation, the relative increase of risk incidence of premature SGA in two- (combined with exposure of SO_2_) and three-pollutant (combined with exposure of SO_2_ and NO_2_) models were 53% (95% CI: 1.38–2.47) and 73% (95% CI:1.18–2.57) after adjustment for the potential confounders. Further, we examined the associations between PM_2.5_ concentrations and premature SGA in different exposure levels, as Table 5 shown, the OR (95% CI) of premature SGA increasing across quintiles of PM_2.5_, SO_2_, NO_2_ concentrations during the whole gestation period adjusting for confounders (*P* for trend < 0.001). The restricted cubic spline regression model was used to evaluate the associations continuously (Table 5) and the regression splines suggested a possible threshold effect for PM_2.5_ concentrations of approximately 40 μg/m^3^ on premature SGA risk, for SO_2_ and NO_2,_ the effect became flat at approximately 45 and 35 μg/m^3^, and these air pollutants concentrations showed linear relation with premature SGA risk.

**Table 4:** The estimated ORs (and 95% CIs) of SGA associated with estimated exposure levels during exposure period

Air pollutants and model ^a^	Trimester1			Trimester2			Trimester3			Whole gestation		
	OR ^b^	95% CI		OR ^b^	95% CI		OR ^b^	95% CI		OR ^b^	95% CI	
PM_2.5_ (μg/m^3^)												
Single-model	1.01	0.47	3.02	1.34	0.67	1.98	1.09	0.67	1.86	1.03	0.67	1.31
+ SO_2_	1.66	0.62	1.77	1.13	0.96	1.72	1.18	1.03	2.83	1.63	1.38	2.57
+ NO_2_	1.37	0.53	1.86	1.26	0.68	1.98	1.03	0.53	1.26	1.24	0.74	2.17
+ SO_2_ + NO_2_	1.22	0.77	2.99	1.37	0.80	3.54	1.37	1.03	3.58	1.83	1.18	2.97
SO_2_ (μg/m^3^)												
Single-model	1.01	0.58	2.82	1.04	0.75	1.57	0.99	0.61	1.97	1.00	0.94	1.25
+ PM_2.5_	0.99	0.74	3.78	0.98	0.76	1.52	0.96	0.67	0.99	0.95	0.89	1.47
+ NO_2_	1.02	0.59	1.26	1.03	0.84	1.83	0.99	0.48	1.98	0.99	0.84	1.57
+ PM_2.5_ + NO_2_	1.03	0.74	3.31	1.10	0.95	1.52	1.17	0.76	2.71	1.05	0.76	2.99
NO_2_ (μg/m^3^)												
Single-model	1.04	0.80	2.97	1.24	0.58	1.67	1.00	0.92	1.41	1.00	0.67	1.94
+ PM_2.5_	0.99	0.52	1.34	1.01	0.94	1.95	0.99	0.89	1.46	0.99	0.92	1.57
+ SO_2_	1.26	0.66	1.82	1.01	0.94	2.97	1.01	0.95	2.17	1.14	0.88	2.18
+ PM_2.5_ + SO_2_	1.31	0.72	3.83	1.26	0.93	3.26	0.99	0.79	2.85	1.23	0.67	2.56

SGA: small for gestational age, OR: odds ratio, CI: confidence interval, PM_2.5_: particular matter, NO2: nitrogen dioxide, SO2: sulphur dioxide

a. Adjusting for infant gender, maternal age (< 20, 20–30, 30–40, > 40) and parity (once, twice or more), year of birth (2013, 2014, 2015 and 2016), season of birth (spring, summer, autumn, winter), gestational age, and meteorological information (mean temperature, relative humidity and air pressure)

b. OR per 10 units increase

**Table 5:** The adjusted ORs (and 95% CIs) of SGA according to the estimated exposure levels in three-pollutants model, for trimester specific exposure windows, among 2709 preterm births

Air pollutants levels ^a^	NO.	Trimester1			Trimester2			Trimester3			Whole gestation		
		OR ^b^	95% CI		OR ^b^	95% CI		OR ^b^	95% CI		OR ^b^	95% CI	
PM_2.5_ (μg/m^3^)													
1st quartile	677	1.00	–	–	1.00	–	–	1.00	–	–	1.00	–	–
2nd quartile	677	1.03	0.76	1.87	0.83	0.56	1.34	1.32	0.80	2.18	1.05	0.56	1.64
3rd quartile	677	1.13	0.87	2.13	1.28	0.65	1.67	1.46	0.87	2.29	1.41	0.44	1.15
4th quartile	678	1.35	1.02	2.56	0.97	0.76	1.43	1.91	1.21	2.83	2.33	1.02	2.68
*P for trend*		0.121			0.374			0.025			0.001		
NO_2_ (μg/m^3^)													
1st quartile	677	1.00	–	–	1.00	–	–	1.00	–	–	1.00	–	–
2nd quartile	677	1.01	0.68	1.58	0.99	0.63	1.56	1.16	0.76	1.98	0.96	0.54	1.47
3rd quartile	676	1.08	0.71	1.62	1.30	0.79	2.01	1.06	0.65	1.78	0.98	0.69	1.87
4th quartile	679	0.87	0.53	1.33	1.36	0.89	2.31	1.45	0.91	2.23	1.06	0.54	2.92
*P for trend*		0.436			0.076			0.165			0.016		
SO_2_ (μg/m^3^)													
1st quartile	677	1.00	–	–	1.00	–	–	1.00	–	–	1.00	–	–
2nd quartile	677	1.17	0.89	1.83	1.20	0.77	1.38	1.01	0.53	1.17	0.98	0.89	1.15
3rd quartile	677	1.05	0.99	1.11	1.06	0.89	1.23	1.24	0.78	1.32	1.02	0.82	1.38
4th quartile	678	1.01	1.01	1.25	1.12	0.88	2.41	1.15	0.89	2.56	1.18	0.77	2.51
*P for trend*		0.657			0.175			0.078			0.004		

SGA: small for gestational age, OR: odds ratio, CI: confidence interval, PM_2.5_: particular matter, NO_2_: nitrogen dioxide, SO_2_: sulphur dioxide

a. Adjusting for infant gender, maternal age (< 20, 20–30, 30–40, > 40) and parity (once, twice or more), year of birth (2013, 2014, 2015 and 2016), season of birth (spring, summer, autumn, winter), gestational age,, and meteorological information (mean temperature, relative humidity and air pressure)

b. OR per 10 units increase

## Discussion

To our knowledge, this is the first study to focus on the relationship between air pollution and premature SGA. The IDW model were applied to evaluate precisely the air pollutant concentrations (PM_2.5_, SO_2_ and NO_2_), and this retrospective cohort study explored the adverse effects of maternal exposure on fetus outcomes in Changzhou, 2013–2016. The results indicate that increased PM_2.5_ levels were significantly associated with higher risks of premature SGA in multi-pollutants models in the third trimester and whole gestational period.

Changzhou is a modern city but less industrialized city. Its air pollution possesses unique components, so the similar studies from other cities or regions cannot replace the local study like this one in Changzhou. We collected the pregnant data from the City Maternal and Child Health Care Hospital, which receives half of the delivery women in the city and could fairly represent the local situation.

In this study, the incidence of premature SGA and air pollutants concentration increased by years in Changzhou, 2013–2016. A significant positive association was identified between air pollution and premature SGA, which is in accordance with many previous studies [23, 24]. Then we estimated individual exposure to PM_2.5_, SO_2_ and NO_2_ in each trimester as well as over the whole pregnancy. The data of pollutant values were interpolated into trimester-specific and entire pregnancy. By generalized estimating equations regression models, gestational exposure to PM_2.5_ combined with SO_2_ or SO_2_ + NO_2_ increased SGA prevalence by 18 and 37% per 10 μg/m^3^ increments during the third trimester, respectively. As we know, embryonic development has sensitive windows to harmful environmental substances. In the early stage of development, if the pregnant mother is exposed to pollutions, it can likely result in abortion, stillbirth or deformity [25]. In the first trimester, the exchange of substance between embryo and mother is relatively low, and the inner exposure dose of air pollutants is low, therefore, the harmful effects from exposure would be less severe. While in the third trimester when the fetus develops fast, plenty nutrition, as well as toxic substrates, was delivered to embryo for its rapid growth, so the internal exposure dose increased, the hazard effects may be amplified, and in this period, the toxic effects might show as hypoevolutism [26].

We observed that both prevalence of SGA and the mean concentrations of pollutants (PM_2.5_, SO_2_ and NO_2_) are higher in winter and lower in summer, which was in consistent with meteorology that higher temperature facilitates cyclone and is beneficiary for the pollutants to diffuse. And women with premature fetus were those who start pregnant in winter when the air pollutants concentrations were higher. It should be noted that the above difference was not significant in this study, which might be attribute to the sample size. Further studies on this would be warranted. Air pollutants were reported to induce premature under many molecular mechanisms, including causing maternal inflammation [27], affecting placenta development [28] or inducing ROS-derived damage [29], but the mechanisms for air pollution-related premature SGA need to be further studied.

Air pollutants were complex, and could have combinational toxic effects (Šaueret al., 2018), pollutants could alter the chemical properties and enhance the toxicity of each other. For instance, PM_2.5_ could absorb other toxic substance and enhance their toxicity. That might explain that why there was no significant association between PM2.5 and SGA in a single model but a significant association in multi-pollutant mode in our study. Therefore, we analyzed the interaction between different pollutants, and the results showed that PM_2.5_ exhibits an increased risk when SO_2_ and/or NO_2_ were a combined risk(s), which was similar to the previous studies [30]. Our study also showed that PM_2.5_ might be the main risk factor for premature SGA, and we should set priority to low its concentration in the polluted air.

Strengths of the study included the retrospective cohort study and the accurate IDW exposure method based on the coordinate of monitoring stations and individual residential locations. However, the time spends in traffic, occupational activities and moving during pregnancy were unavailable. Although a series of potential confounders were controlled, maternal smoking and alcohol consumption, known risk factors for premature births, were excluded in this model. The main reason was that we focused on the pregnant consumption of tobacco and alcohol in every trimester pregnancy during the whole gestational period, and none was exposed. The maternal disease is also associated with premature, however, which was not adjusted for in our models since information on maternal diseases was missing for a large number of pregnant women. On the other hand, amount of important covariates was taken into account: delivery, gestational age, parity, natural delivery or not, sex of infants, birth years, birth season of birth. In addition, the positive association does not mean causality in a retrospective study.

## Conclusion

In this retrospective study, we confirmed the association between premature SGA and air pollution in Changzhou city, and found that the third trimester was the window of susceptibility for the premature birth weight. We analyzed and identified the risk of several air pollutants, as well as their interactive effects, and indicated that PM_2.5_ contributed more risk to the premature SGA than SO_2_ and NO_2_.

## Data Availability

Data used for analysis is available upon a proper request from the corresponding author.

## Abbreviations

AGA: Appropriate for gestational age
CI: Confidence interval
GEE: Generalized estimating equations
NO_2_: Nitrogen dioxide
OR: Odds ratio
PM: Particulate matter
SGA: Small for gestational age,
SO_2_: Sulfur dioxide
WHO: World health organization
